# Stem Loop Sequences Specific to Transposable Element *IS605* Are Found Linked to Lipoprotein Genes in *Borrelia* Plasmids

**DOI:** 10.1371/journal.pone.0007941

**Published:** 2009-11-20

**Authors:** Nicholas Delihas

**Affiliations:** Department of Molecular Genetics and Microbiology, School of Medicine, State University of New York, Stony Brook, New York, United States of America; Institut de Pharmacologie et de Biologie Structurale, France

## Abstract

**Background:**

Plasmids of *Borrelia* species are dynamic structures that contain a large number of repetitive genes, gene fragments, and gene fusions. In addition, the transposable element *IS605/200* family, as well as degenerate forms of this *IS* element, are prevalent. In *Helicobacter pylori*, flanking regions of the *IS605* transposase gene contain sequences that fold into identical small stem loops. These function in transposition at the single-stranded DNA level.

**Methodology/Principal Findings:**

In work reported here, bioinformatics techniques were used to scan *Borrelia* plasmid genomes for *IS605* transposable element specific stem loop sequences. Two variant stem loop motifs are found in the left and right flanking regions of the transposase gene. Both motifs appear to have dispersed in plasmid genomes and are found “free-standing” and phylogenetically conserved without the associated *IS605* transposase gene or the adjacent flanking sequence. Importantly, *IS605* specific stem loop sequences are also found at the 3′ ends of lipoprotein genes (PFam12 and PFam60), however the left and right sequences appear to develop their own evolutionary patterns. The lipoprotein gene-linked left stem loop sequences maintain the *IS605* stem loop motif in orthologs but only at the RNA level. These show mutations whereby variants fold into phylogenetically conserved RNA-type stem loops that contain the wobble non-Watson-Crick G-U base-pairing. The right flanking sequence is associated with the family lipoprotein-1 genes. A comparison of homologs shows that the *IS605* stem loop motif rapidly dissipates, but a more elaborate secondary structure appears to develop in its place.

**Conclusions/Significance:**

Stem loop sequences specific to the transposable element *IS605* are present in plasmid regions devoid of a transposase gene and significantly, are found linked to lipoprotein genes in *Borrelia* plasmids. These sequences are evolutionarily conserved and/or structurally developed in an RNA format. The findings show that *IS605* stem loop sequences are multifaceted and are selectively conserved during evolution when the transposable element dissipates.

## Introduction

*Borrelia* species are unusual in that they carry a large number of plasmids [1], [2]. Many of these plasmids encode a heterogeneous group of lipoproteins, some of which are outer cell surface proteins. Several lipoprotein genes are in a state of flux whereby plasmids encode multiple gene copies that vary in sequence and encode fragments of these genes as well [2]. Transposable elements are also present, along with fragmented and degenerate transposase (Tpase) genes. Short repeat sequences are also found, as well as stem loop sequences that are linked to virulence protein genes [3], complement regulator-acquiring surface protein 1 (CRASP_1) and lipoprotein_1 (part of the paralogous gene family termed PFam60). The recent availability of complete genomic sequences from multiple *Borrelia burgdorferi* strains as well as different strains of *Borrelia garinii* and *Borrelia afzelii* (see http://www.ncbi.nlm.nih.gov/sutils/genom_table.cgi) allows for a detailed phylogenetic comparison between strains, which has heretofore not been available.

In a series of elegant studies, the transposable element *IS605/IS200* family in *Helicobacter pylori*, termed ISHp608 (*IS608*) was shown to display a DNA sequence that folds into a small stem loop structure. This stem loop is found in both the left (upstream) and right (downstream) flanking regions of the Tpase. The stem loop plays an integral role in transposition as it serves as a recognition site for the *IS608* transposase and binds the Tpase prior to catalytic cleavage of single stranded DNA [4]–[6]. Here we show that the transposable element (TE) *IS605* (*IS605B*) in *Borrelia spp.* also displays sequences in flanking regions that fold into stem loops, however the upstream and downstream motifs differ. These motifs are found evolutionarily conserved in intergenic spaces outside the context of the TE, and importantly, they are also found associated with the 3′ ends of lipoprotein genes. It should be noted that the *Borrelia IS605B* TE has been recognized before and was termed PFam82 [2].

## Results

### Characterization of *IS605B* Flanking Regions

The *IS605B* (PFam82) sequence from gene locus BBU29805_Z02 in *B. burgdorferi strain 29805* was used as a model. This is a simple transposable element that carries only the transposase gene. However, it should be considered a putative TE as it has not yet been experimentally shown to transpose. The *IS605B* Tpase gene, which encodes a 377 amino acid (aa) polypeptide is usually present in one or two full-length copies in *B. burgdorferi* plasmid genomes, but fragments and degenerate sequences are common. Full-length copies of *IS605B* are found in four other *Borrelia* species (*B. afzelii*, *B. garinii*, *B. duttonii*, *B. recurrentis*), but *Borrelia sp. SV1* lacks a complete copy and translated sequences show only polypeptides of a fragmented *IS605B* Tpase (i.e., they contain an mRNA with a premature stop codon) and degenerate Tpase sequences. The original genome sequence of *B. burgdorferi* strain B31 showed no intact PFam82 but the newly released *B. burgdorferi* genomes often have one or two intact copies.

**Figure 1:**
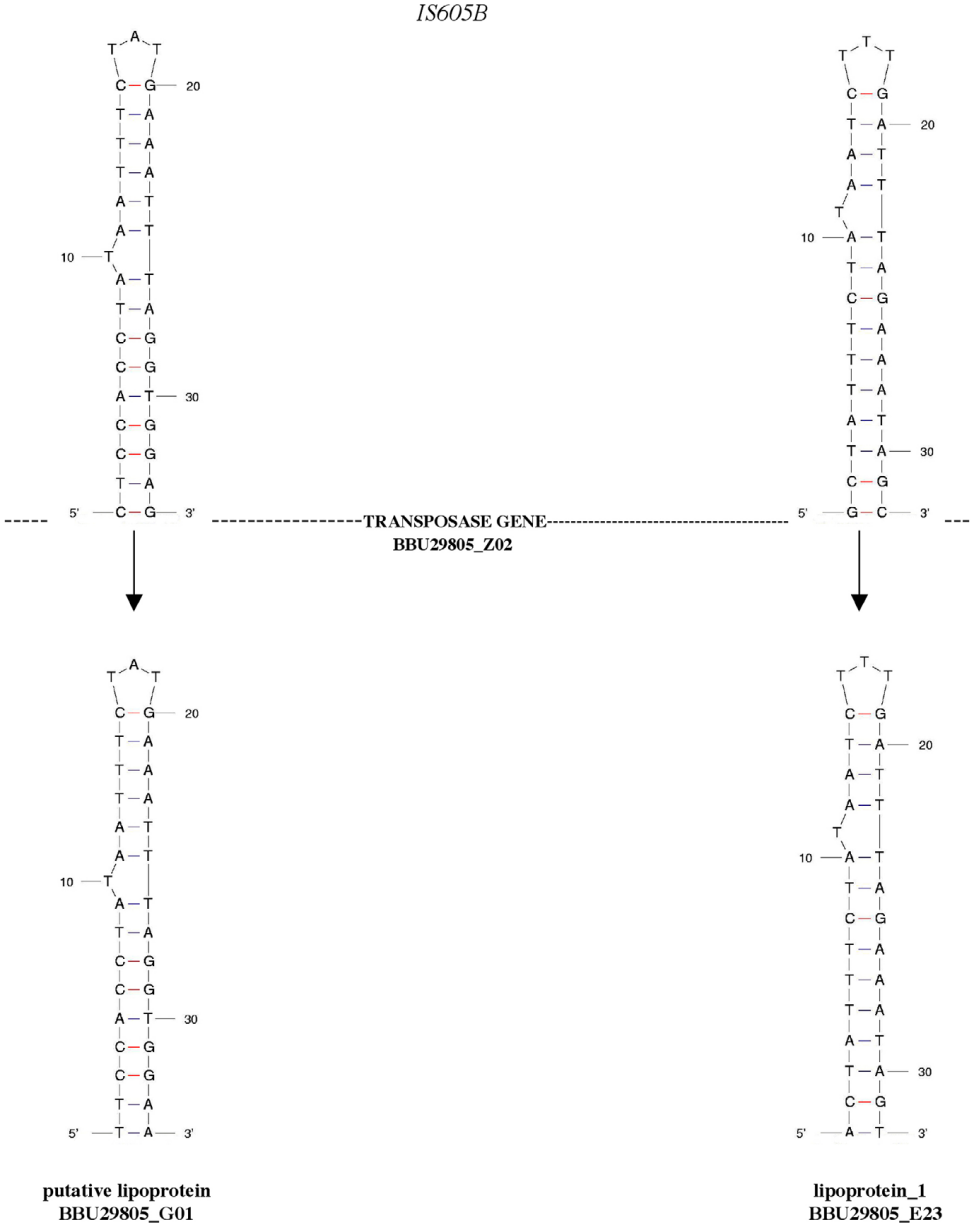
Stem loop structures. (Top figure). A schematic of the *IS605B* element from *B. burgdorferi 29805*, plasmid 29805_lp28-2 (GenBank accession no. NC_012502) showing the left and right flanking region stem loops. The end of the left flanking region of *IS605B* extends 25 bp beyond the 5′ end of the left stem loop. The end of right flanking region of *IS605B* is 9 bp from the right stem loop 3′ end. (Bottom figure). Stem loops found at the 3′ end regions of lipoprotein (BBU29805_G01) and lipoprotein_1 (BBU29805_E23).

A schematic of the BBU29805_Z02 *IS605B* structure is shown in Figure 1. To find the putative ends of the *IS605B* flanking regions, 100 nt of the Tpase coding region, together with 200 nt upstream or 200 nt downstream of the Tpase gene were used to blast *Borrelia* genomes for homologous sequences. The identity between homologous sequences of *IS605B* from different *Borrelia spp*. drops precipitously at position –108 nt upstream from the Tpase AUG (ATG) start codon (left side) and 117 nt downstream of the stop codon (right side). These positions most likely define the ends of the *IS605B*.

Secondary structure modeling of the left and right flanking sequences of *IS605B* from locus BBU29805_Z02 reveals that segments of these sequences can fold into stem loops with specific motifs that contain a bulged T on the 5′ side of the stem (Figure 1, top). Left and right stem loops differ in location of the bulged T within the stem, as well as in the type of base-pairing. Stem loop motifs are highly conserved between *IS605B* elements of *Borrelia spp*, but they display some structural differences from those of the *H. pylori IS608* stem loop. *IS608* has the identical stem loop structure in both the right- and left- flanking regions, has a bulged T on the 3′ side of the stem, and displays a shorter stem compared to the *IS605B* stem loops [6]. However the left *Borrelia* stem loop does have the signatures found in *Helicobactor* that are important for Tpase recognition, i.e., a bulged T at position 10 and a T residue at the loop position 17. These two T residues are in the identical position on the polynucleotide chain relative to each other, as in the case in the *Helicobacter IS 608* structure, i.e., they are separated by 6 nucleotides. Both the bulged T_10_ and T_17_ of the loop are crucial for Tpase binding in *Helicobacter* [4].

### *IS605B* Stem Loops Associated with Lipoprotein Genes

*IS605B* flanking stem loop sequences were used to scan *Borrelia* plasmid genomes for homologous sequences outside of regions that have the *IS605B* transposase gene or Tpase gene fragments. Both stem loop sequences are found “free-standing” in plasmid integenic regions, i.e., without an accompanying *IS605B* Tpase gene sequence or fragments. Surprisingly, in addition to their presence in areas devoid of open reading frames, the two motifs are also found associated with the 3′ ends of some lipoprotein genes. Stem loop motifs associated with the putative lipoprotein gene locus BBU29805_G01 and the lipoprotein_1 from BBU29805_E23 are shown as examples (Figure 1, bottom). The putative lipoprotein gene has a translated aa sequence that displays a signal peptide sequence, and lipoprotein_1 has the domains of the *Borrelia*_lipo_1 super family. However It should be noted that BBU29805_G01 is homologous to PFam12 members and BBU29805_E23 is homologous to PFam60 members. The *IS605B* left and right stem loop motifs are specific to each class of lipoprotein genes and show identical stem loops to those displayed by *IS605B*, with the exception of one base-pair compensatory change at the base of each stem (Figure 1).

**Figure 2:**
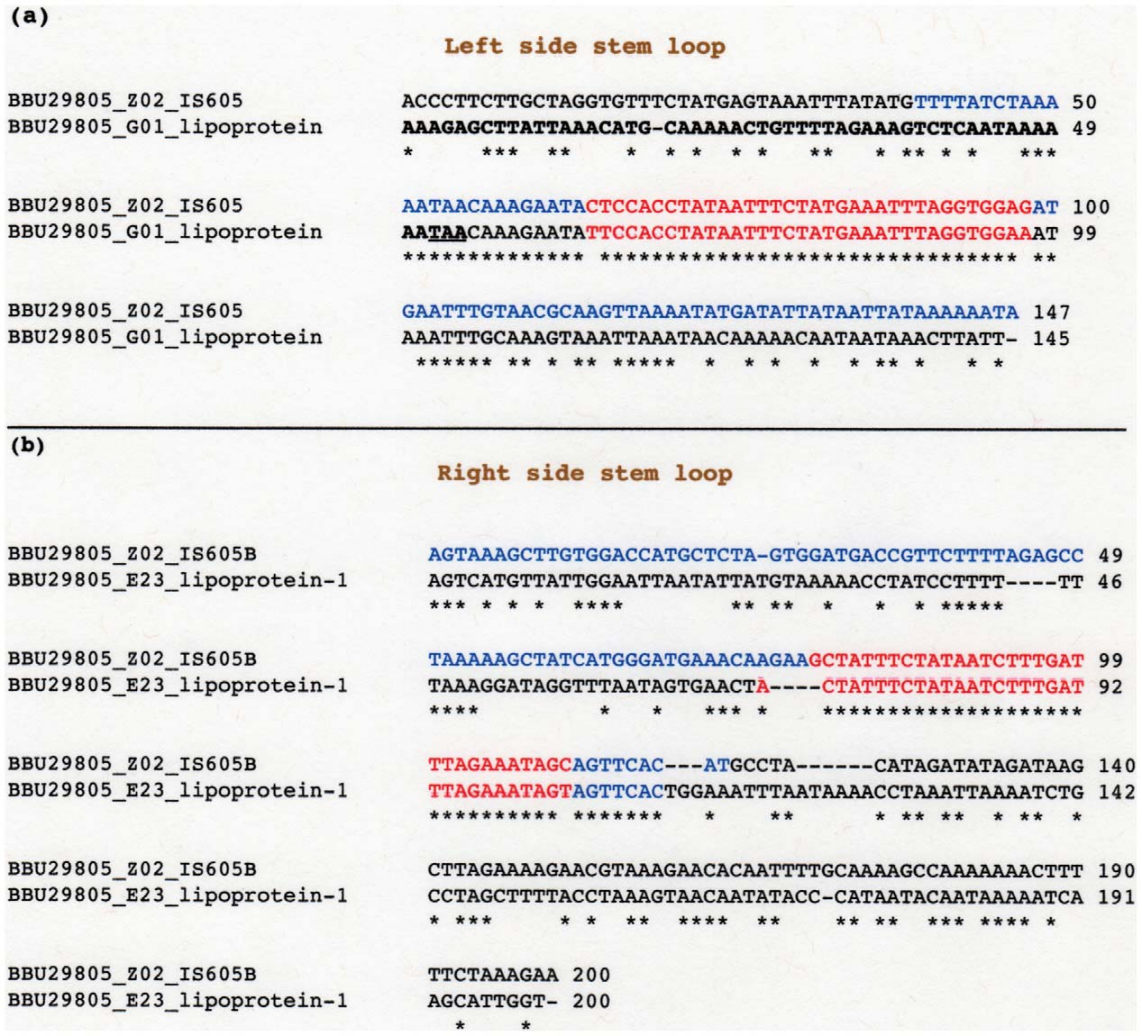
Nucleotide sequence alignments. (a) Alignment of nt sequences from the coding and the downstream region of BBU29805_G01 with the *IS605B* left flanking sequence. (b) Alignment of nt sequences downstream from the stop codons of BBU29805_E23 and *IS605B* Tpase, which comprises the right flanking sequence. Left and right *IS605B* flanking sequences are shown in blue. Stem loops are in red. Alignments were by the ClustalW2 program (http://www.ebi.ac.uk/Tools/clustalw2/index.html).

Alignment of nucleotide sequences downstream from the two classes of lipoprotein genes with sequences from the left and right flanking regions of *IS605B* is in Figure 2. The left side *IS605B* sequence (Figure 2a) represents 147 base pairs upstream from the Tpase AUG (ATG) translational start site start. The *IS605B* left flanking region is shown in blue. The sequence extends beyond the flanking region, which ends at position 39 (–108 from the AUG initiator codon). Sequences shown for the lipoprotein gene locus BBU29805_G01 contain 55 nt of the 3′ end coding sequence. These coding sequences are shown in black and bold lettering. The TAA (UAA) stop codon is underlined. Sequences downstream of the lipoprotein stop codon are also shown, and this region contains the left stem loop sequence (in red). The alignment shows a near perfect identity between the stem loop sequences and the region 5′ to the stem loop, which includes 8 nt of the lipoprotein coding region (to position 47). A high similarity between the two sequences also exists 23 nt past the 3′ end of the stem loop sequence, up to position 120 of the lipoprotein gene downstream sequence. The homology drops off downstream of this region, which indicates that this section of the *IS605B* left flanking sequence is not included in the lipoprotein gene downstream region.

Important is the overlap in similarity between the last 8 nt of the coding region of BBU29805_G01 and the *IS605B* flanking sequence. Thus a small section of the *IS605B* flanking sequence forms part of the 3′ end coding region of the BBU29805_G01 lipoprotein gene. This type of overlap is similar to that of several inverted repeat sequences found in other bacteria, whereby the inverted repeat sequence also overlap the 3′ ends of coding regions of adjacent genes [7], [8].

To assay for possible remnants of the Tpase aa sequences, 1000 nt downstream of the stop codon of locus BBU29805_G01 were used in a blastx search. No aa sequences that correspond to fragments or degenerate sequences of the transposase were detected, and only the aa sequence of the gene downstream of BBU29805_G01, BBU29805_G02, a putative protein adenine specific DNA methyltransferase was found. In addition, a blastn search with the 1000 nt downstream sequence did not reveal the presence of nt sequences homologous to the *IS605B* right flanking nt sequence. Thus only the *IS605B* left stem loop sequence, with short adjacent sequences on the 5′ and 3′ ends of the stem loop are found linked to the BBU29805_G01 lipoprotein gene.

Alignment of the right side flanking sequence of *IS605B* (shown in blue), with the downstream sequence from gene locus BBU29805_E23 lipoprotein_1 (in black) is in Figure 2b. Both sequences represent 200 bp downstream of their respective the stop codons. The alignment shows a perfect identity between the stem loop sequences (in red), with the exception of the two end nt. A high sequence similarity persists up to 7 nt downstream of the stem loop (to position 117 of *IS605B*). The identity drops off sharply in sequences immediately 5′ of the stem loop. Thus the *IS605B* right stem loop sequences, including 7 bp downstream of the stem loop are the only *IS605B* sequences associated with locus BBU29805_E23 lipoprotein_1.

The role(s) of stem loops associated with lipoprotein genes is unknown, but they may function at the DNA level, possibly as recognition sites for Tpase. However a functional role at the RNA level should also be considered. Repeat sequences in other bacteria, some of which are the non-autonomous miniature inverted repeat transposable elements (MITEs), fold into stem loops and are found linked to protein genes. These are transcribed into RNA and are thought to serve as regulatory elements [9]–[11]. In the *Borrelia* lipoprotein genes described here, paralogs and orthologs of these genes show that stem loop sequences downstream of the lipoprotein genes have mutated and display G-U non-Watson-Crick base pairing in their stems. In structure modeling of these sequences, phylogenetic conservation of the secondary structure motif is maintained at the RNA level, but not necessarily at the DNA level, and some DNA sequences show unfolded structures. Thus a comparison of stem loop orthologs and paralogs are presented in the RNA form.

### Putative Lipoprotein Gene Paralogs (PFam12 Family)

**Figure 3:**
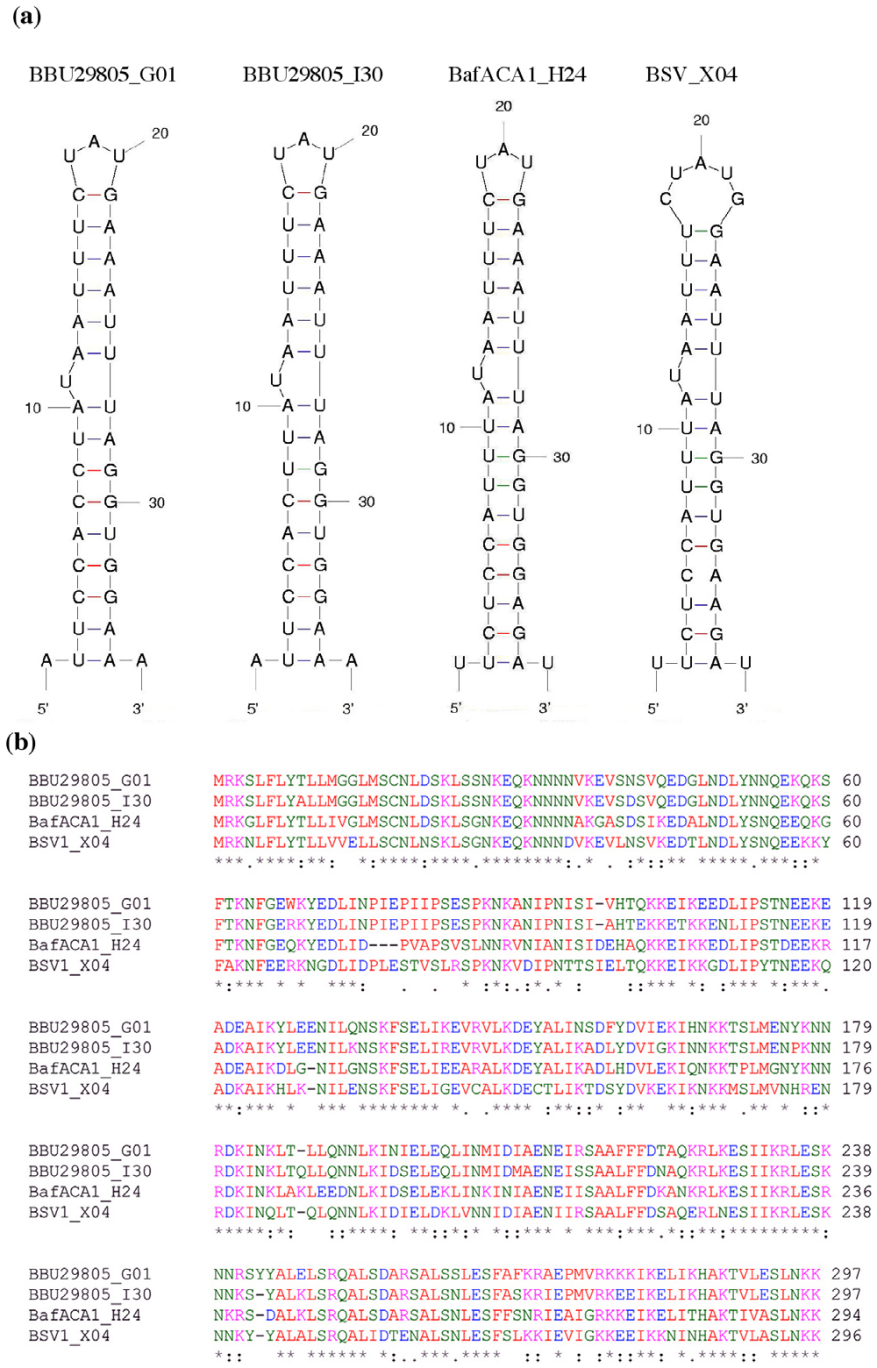
RNA secondary structure models and amino acid sequence alignments. (a) Secondary structure models of stem loop sequences downstream of *Borrelia spp* lipoprotein genes. To find homologous lipoprotein genes, the BBU29805_G01 aa sequence was used in an NCBI protein blast search using the blastp algorithm, protein-protein BLAST (website http://blast.ncbi.nlm.nih.gov/Blast.cgi). Identified lipoprotein gene homologs were then scanned for stem loop sequences in their downstream regions. The Expect values using the BBU29805_G01 aa sequence in blast searches are: Expect = 9e−143 for BBU29805_I30, Expect = 3e−108 for BafACA1_H24, Expect = 1e−100 for and BSV_X04 (b) Alignment of amino acid sequences from lipoprotein genes. BBU29805_G01 and BBU29805_I30 are paralogs. BafACA1_H24 and BSV_X04 are orthologous to the *B. burgdorferi str. 29805* genes. The ClustalW2 program was used for alignment (Labarga et al., 2007). Amino acid color code: red, hydrophobic and aromatic amino acids, blue, acidic, magenta, basic, green, hydroxyl and amine containing as specified by the EMBL-EBI CLUSTALW 2.0.8 multiple sequence alignment program. Symbols used according to the ClustalW2 program: (*) denotes invariant amino acid positions, (:) denotes conserved substitutions, (.) denotes semi-conserved substitutions.

Figure 3a shows RNA structural models of two paralogous sequences from *B. burgdorferi str. 29805* and related homologs from *B. afzelii ACA-1 and Borrelia sp. SV1*. The stem sequences show several mutations, but the stem loop motif is maintained via G-U base pairing (compare stem structures) and a base-pair compensatory change, i.e., U_2_-A_35_ (BBU29805_G01) and C_3_-G_36_ (BafACA1_H24). The BafACA1_H24 and BSV_X04 structures have an additional pairing at the base of the stem, which extends the number of base-pairs from 15 to 16.

An alignment of translated aa sequences from the four lipoproteins show that coding sequences have diverged (Figure 3 b). For example, there is 73% aa identity (resultant E = 3e−108) between BBU29805_G01 and BafACA1_H24 protein sequences. However the stem loops, which have also sustained mutations, remain thermodynamically stable (delta G of −15.8 kcal/mol vs −14.0 kcal/mol) and the motif is unchanged. BSV_X04 does show mispairing at C_5_ and A_34_ and breathing at the top loop, but the sequence still retains the bulged U motif and folds into a stable stem loop (delta G = −6.8 kcal/mol). DNA secondary structure modeling shows an unfolded stem loop for the BSV_X04-associated sequence and a total loss of stem loop signatures (Figure S1). A search for a homolog to lipoprotein BBU29805_G01 in *B. garinii strains* yielded only a distantly related gene at locus BGAFAR04_K0008. This gene has only 49% identity to BBU29805_G01 and does not display the stem loop sequence in its downstream region. Homologs to BBU29805_G01 have not been found in other *Borrelia spp*. other than those mentioned above.

The paralogous genes, BBU29805_G01 and BBU29805_I30 in *B. burgdorferi 29805* have different genes in their immediate downstream regions, i.e., BBU29805_G02, a putative adenine specific DNA methyltransferase and BBU29805_I29, a putative type II restriction enzyme, respectively. The two paralogs, BBU29805_G01 and BBU29805_I30 also show little similarity in intergenic sequences downstream of their stem loops (data not shown). Thus the paralogs differ in the type of gene that is in the immediate downstream region and in the downstream intergenic sequences. Nevertheless they have nearly identical stem loop sequences. This is significant in that the stem loop sequences are highly conserved, yet the adjacent downstream region is totally different.

As there was a duplication of the lipoprotein gene in *B. burgdorferi 29805*, it is possible that the BBU29805_G01 was duplicated and inserted at the BBU29805_I30 genomic site, but that only BBU29805_G01 and its associated stem loop sequence was transferred. As the stem loop of BBU29805_G01 has the *IS605B* motif with the T residue signatures for Tpase recognition at the DNA level, the possibility remains that the stem loop DNA sequence of BBU29805_G01 participated in this putative duplication of BBU29805_G01.

Evolutionarily conserved stem loop sequences were previously subjected to random mutations, for example, sequences of stem loop 1 (described below) [3]. To determine the probability that BafACAI_H24- and BSV_X04-associated stem loop structures could arise by chance via mutations in the BBU29805_G01-linked stem loop sequence, random mutations were introduced into the BBU29805_G01 RNA-type stem loop sequence. Two hundred random mutagenesis trials were performed, each for 6 and 8 base changes (the number of base residues changed per 38 nt sequence for BafACAI_H24 and BSV_X04, respectively). The Stormoff mutagenesis program was used to induce random mutations (http://molbiol.ru/eng/scripts/01_16.html). No structures that show the exact secondary structural features of the homologs were generated. However two out of 200 mutated sequences display secondary structures similar to BafACAI_H24 and five out of 200 show similarities to BSV_X04. The major similarity involves the bulged U at the precise position in the stem, but on the other hand these mutated structures do not display several other signatures, e.g., the U residue in the loop. We conclude the probability that sequences produced by random mutations would generate identical structures in homologs is less than 0.005, but that 1% and 2.5% respectively, of each of 200 random mutagenesis trials show a bulged U residue in the identical location to that of the presumed parent stem loop sequence linked to BBU29805_G01.

### Lipoprotein-1 Gene Homologs (PFam60 Family)

**Figure 4:**
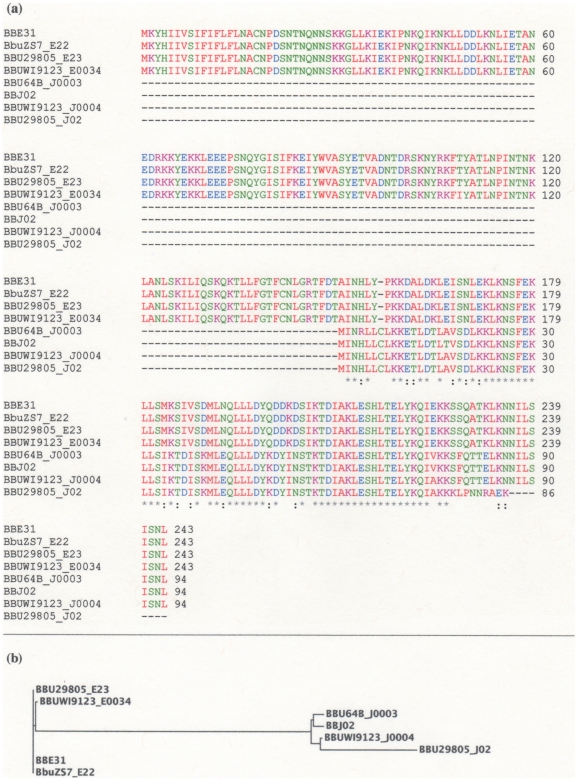
Amino acid sequence alignments and phylogram. (a) Alignment of amino acid sequences from eight lipoprotein-1 genes. The ClustalW2 program was used. The color code and symbols shown in the figure are as described in Figure 3b. Homologs of lipoprotein-1 gene BBU29805_E23 were found by a protein blast search of *B. burgdorferi 29805* strains using the blastp algorithm, protein-protein BLAST (website http://blast.ncbi.nlm.nih.gov/Blast.cgi). The aa sequence of BBU29805_E23 was used in the search to find paralogous genes. The four full-length homologs have an identity of 99–100% relative to BBU29805_E23. The identity of the four truncated paralogs relative to BBJ_02 is 97%. The identity between BBU29805_E23 and BBJ_02 is 73% with E = 3e−25. (b) Phylogram of the eight lipoprotein_1 amino acid sequences. Phylogram shown is as determined by the ClustalW2 program. The branch lengths of the tree are proportional to the degree of evolutionary change.

Eight genes related to the super family of lipoprotein_1 genes (Pfam60) found in *B. burgdorferi* strains have been found to display stem loop motifs in their downstream sequences. These lipoprotein_1 genes branched out into two classes. An alignment of translated aa sequences is in Figure 4a. One class encodes a full- length 243 aa protein, and another a fragment of the C-terminal end which consists of 94 aa. The 243 aa and the 94 aa species each are found in four *B. burgdorferi* strains and they have developed their own specific sequences. A phylogram of aligned aa sequences shows a clear separation into two classes (Figure 4b). This is based on aa sequence signatures as opposed to size, as a phylogram of only the C-terminal 94 aa peptides of all eight proteins reveals the same pattern (data not shown). Both the full length and 94 aa subfamilies are each found on the same respective *B. burgdorferi* plasmids, i.e., lp25 for the 243 aa full length and lp38 for the 94 aa species, and they are found in similar locations on these plasmids.

**Figure 5:**
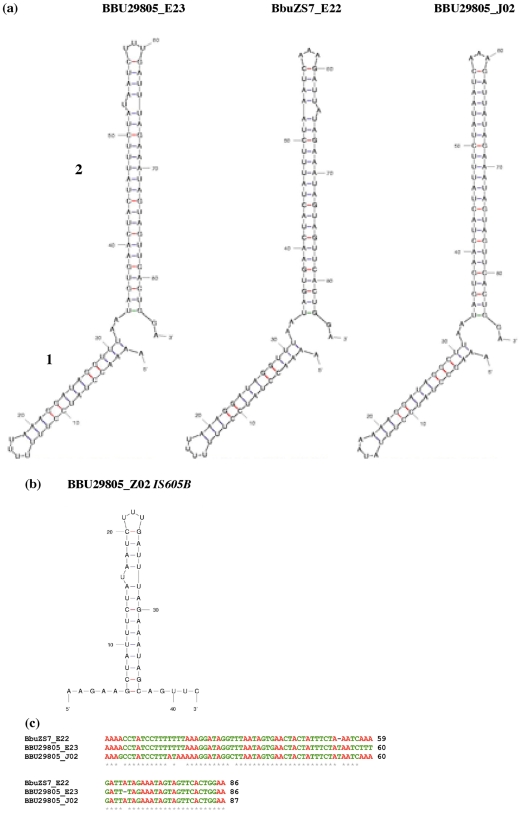
RNA secondary structure models and nucleotide sequence alignments. (a) Secondary structure models of sequences downstream of lipoprotein_1 genes from three representative genes. Position A_1_ represents 30 nt downstream of lipoprotein_1 mRNA stop codons. The mfold program was used to fold RNA sequences. (b) *IS605B* stem loop in RNA form. (c) Alignment of nt sequences shown in the secondary structure models. The alignment includes an addition position at the 3′ end. Adenosine nucleotides are in red for ease of viewing of the alignment. Dots under the sequences represent invariant positions. Alignment was by the ClustalW2 program.

All eight lipoprotein_1 genes show sequences in the region downstream of the stop codon that fold into two stem loops. Figure 5a shows representative examples. The 5′ side secondary structure (stem loop 1) shows a highly conserved 11–13 base-paired stem loop with conserved adenosine residues straddling the base. This structure was previously reported to be located downstream of the family lipoprotein_1 genes and shown to be phylogenetically conserved with multiple base-pair compensatory changes and base-pair transversions as well [3]. Stem loop 2 is on the 3′ side of the stem loop 1 structure. The BBU29805_E23 associated stem loop 2 has the signatures of the BBU29805_Z02 *IS605B* right flanking stem loop. The *IS605B* stem loop is shown in RNA form for comparisons (Figure 5b).

The *IS605B*-type stem loop appears to have rapidly evolved within the *B. burgdorferi* strains. All homologs display extended stem loops e.g., BBU29805_E23 has 23 base pairs in the stem whereas BBU29805_Z02 *IS605B* has 14. There are also specific changes such that the bulged U_53_ of BBU29805_E23 appears deleted and an A inserted at position 63/64 in BbuZS7_E22. The top loop, U_58_UU (BBU29805_E23 numbering) is changed to AAA in BbuZS7_E22 and BBU29805_J02. An A insertion at position 63/64 in BBU29805_J02 (or lack of a U deletion at U_53_) creates a perfect 24 base- paired stem. All four 94 aa lipoprotein genes (Figure 4) display the structure shown for BBU29805_J02 in their downstream regions. Two of the 243 aa lipoprotein genes display the structure for BBU29805_E23 and two the stem loop shown for BbuZS7_E22.

Some of the sequence changes as seen in Figure 5a may be via a putative recombination event whereby the double stranded DNA segment from positions 35–82 (BBU29805_E23 numbering) was placed in the opposite orientation, as the sequence shown for BUZS7_E22 is the same as that of the reverse complement. However we cannot rule out that point mutations alone produced these changes (see alignment Figure 5c). Regardless of the mechanism of change, these mutations are highly specific. The stem is not disrupted but the bulged (U/T) signature is lost in homologs. This is a different pattern of evolution from the stem loop sequence linked to lipoprotein genes (see Figure 3), whereby the *IS605B* stem loop motif is conserved in orthologs (albeit only at the RNA level). It should also be pointed out that the precise positioning of stem loop 2 relative to the 11–13 base-paired stem loop 1 is maintained, and the invariant A residues that straddle the base of stem loop 1 are conserved.

It should be noted that the eight homologs are the genes readily found that show associated stem loops. We do not know if there are more stem loop-associated lipoprotein_1 (Pfam60) genes, ones that perhaps may be more difficult to detect, but further searches and analyses are needed.

### Free-Standing Stem Loops

Both left and right stem loops are found free of an intact *IS605B* element in intergenic regions of *Borrelia* plasmids. Several display base-pair compensatory changes relative to the *IS605B* stem loops. A complete analysis of free-standing stem loops has not been performed, however several representative examples are described here. *B. garinii PBr* plasmid PBr_lp36 has a stem loop at positions 31287-31230. This is in an intergenic region that displays no *IS605B* sequences, Tpase fragments or other protein gene fragments. In *B. burgdorferi 29805* plasmid lp17, there is a free-standing left stem loop sequence located at genomic positions 14511-14561. This is near the 3′ (right) tail end of the plasmid genome where there are no gene annotations. The stem loop shows base-pair compensatory changes relative to the putative *IS605B* parent stem loop. This is the same stem loop sequence that was previously reported in *B. burgdorferi B31*, at genomic positions 14378-14428 but was not originally identified as an *IS* derivative [3]. The 1000 nt sequence downstream of the last annotated gene in *B. burgdorferi 29805* plasmid lp17, the region where the free-standing stem loop is located, appears to only have a highly degenerate 91 aa fragment of a transposase sequence that shows seven stop codons. This sequence is unrelated to *IS605B* but has similarities to the transposase-17 super family proteins.

Sequences similar to the 14 base-paired right flanking *IS605B* stem loop, as well those that have the extended form (see Figure 5, BBU29805_E23 structure) are found free-standing. The exception is the perfect 24 base-paired stem loop (stem loop 2 of BBU29805_J02), which has only been detected as a component of the 94 aa lipoprotein_1 species. An example of the right stem loop [14 base-pairs with a bulged U(T)] is in *B. burgdorferi 29805* plasmid_lp17 at genomic positions 10787-10815. This stem loop sequence is in a 1098 bp integenic region that has a 45 aa degenerate fragment of the 377 aa *IS605B* Tpase (with 65% identity) and a 37 aa fragment of a 140 aa hypothetical protein. An example of a right flanking stem loop in an intergenic region that shows no fragmented Tpase genes or any other fragmented genes is in *B. duttonii Ly* plasmid pl15 at genomic positions 5521-5475.

## Discussion

The functions of stem loops that are downstream of lipoprotein genes are not known. But the BBU29805_G01 and BBU29805_E23 3′ end stem loops retain the *IS605B* flanking region DNA motifs in a stable form and thus potentially may function at the DNA level. Homologs to lipoprotein BBU29805_G01 are likely to function at the RNA level as they have non-canonical base-pairs. Lipoprotein_1 gene homologs have lost the *IS* flanking region stem loop motif and developed elaborate stem loop structures. Stem loops in other prokaryotes are known to function as regulators. For example, MITEs and other inverted repeat elements are found linked to protein genes at either their 3′ or 5′ ends [7], [8], [11], [12]. Some are co-transcribed with the associated protein gene [7], [10]. These transcribed sequences fold into stem loops and are believed to regulate expression of the adjacent genes [10], [11], [13]. By analogy, some of the *Borrelia* lipoprotein-associated stem loops may also serve as regulators.

The two *IS605B* flanking region stem loops are found conserved in plasmid intergenic spaces where the *IS* element is either absent or fragmented. Perhaps a rationale for the selective degradation of the *IS605B* transposase sequence is to create reservoirs of stem loops for further genetic development, yet not overload the genome with additional intact and functional TEs. The concept of reservoirs is not new. Treangen et al. have provided an in-depth discussion of repeat elements in the context of evolutionary reservoirs [14].

Several questions remain. For example, are the stem loops mobile? Repeat sequences in other prokaryotes have been shown to be moved via transposition [7], [11], [15], [16]. Whether the BBU29805_G01 lipoprotein gene-linked stem loop sequence is a remnant of an adjacent *IS605B* sequence that was once intact, or whether the stem loop sequence itself was transferred by transposition or recombination is unknown. With respect to lipoprotein_1 stem loop 2, the exquisite specificity of attachment of stem loop 2 to stem loop 1, whereby the invariant A residue is maintained (see Figure 5) argues against a random process of insertion but suggests a highly restricted one.

How did the extended right stem loop sequence evolve? This may have been via an intermediate process. Specifically, the extended stem loop sequence may have originated in *B. burgdorferi 29805* via horizontal transfer from the *IS605B* upstream (left flanking) sequence of locus BafACA1_F01. This *IS605B* flanking sequence appears to be unique to *B. afzelii ACA-1* plasmid lp28-1, and it displays an aberrant albeit intriguing left upstream flanking region whereby the reverse complement relative to the Tpase mRNA strand folds into three separate stem loops. One stem loop is the same as the one associated with lipoprotein_1 of locus BBU29805_E23, except that it contains 24 as opposed to 23 base pairs. Because they are nearly identical, this BafACA1_F01 *IS605B* stem loop sequence may be the source of the extended stem loop associated with the lipoprotein_1 gene of *B. burgdorferi 29805*, which perhaps was transferred via an intermediate horizontal genetic transfer event. The anomalous left flanking sequence of BafACA1_F01 does have a “normal” copy of the *IS605B* left stem loop, which is identical to that shown in Figure 1. However it also has a perfect 24 base-paired stem (whose sequence also includes ten nucleotides of the 5′ end coding sequence of the transposase gene). This sequence is not related to the *IS605B* left or right flanking stem loop. The complex make up of the anomalous BafACA1_F01 *IS605B* upstream sequence is of interest in itself.

Could the *Borrelia* free-standing stem loop sequences be focal points for formation of new RNA genes? Small regulatory RNAs have been found in *Borrelia* [17], [18] and several RNA-type structures of 120–150 nt with long stem loops have been detected in plasmid intergenic regions [3]. On the other hand, there is new evidence that the sequence of a transposable element may have dual functions. In *Salmonella*, a small non-coding RNA transcript (STnc490) of approximately 80–90 nt has been detected and is encoded on the antisense strand of an *IS200* element [19]. Although this is not associated with an *IS* fragment, it is nevertheless pertinent in that the non-coding RNA transcript sequence overlaps the 5′ end of the transposase gene and the left flanking region of the Tpase gene as well. In addition, the RNA overlaps a stem loop in the left flanking region just 5′ of the Tpase AUG start site. A similar transcriptomic analysis with the use of microarrays [19] would help define small RNA profiles in *Borrelia*, and possibly show *IS* element involvement.

In conclusion, we show for the first time that the transposable element *IS605B* found in *Borrelia spp.* has stem loop sequences that are specific to its right and left flanking regions and that these sequences are also found dispersed in plasmid genomes, either as free-standing units or at 3′ end regions of lipoprotein genes. These dispersed sequences take on their own evolutionary pathways, where mutations preserve the secondary structure at the RNA level and/or the secondary structure is further developed. These findings lead to intriguing questions of function of stem loop sequences as well as the bulged positions at the DNA or RNA levels. Bulged T(U) residues have been shown to be important functional sites in both DNA and RNA stem loops [4], [20], [21]. Fragments of transposase genes are found throughout *Borrelia* plasmid sequences. One rationale for the prevalence of transposable element instability is to provide stem loop sequences for additional uses in the cell.

## Materials and Methods

### Blast Search for Lipoprotein Orthologs and Paralogs

To search for lipoprotein orthologs and paralogs in *Borrelia* strains and species, the amino acid sequences of lipoprotein locus BBU29805_G01 and lipoprotein_1 locus BBU29805_E23 were used in blastp searches of the database (website: http://blast.ncbi.nlm.nih.gov/Blast.cgi) [22]. The General Search Parameters used were as follows: parameters automatically adjusted for short queries; maximum target sequences, 100; the E threshold was 10; word size, 3. The Scoring Algorithm Parameters were: matrix, BLOSUM62; gap costs, existence, 11 extension, 1; compositional adjustments, conditional compositional score matrix adjustment. No filters or maskings were used. The resultant Expect (E) values and percent identities for identified homologs are shown in the text and figure captions.

### Blastn Search for Stem Loop Sequences

To find *IS605B*-related nucleotide sequences near lipoprotein genes in *B. burgdorferi 29805*, blastn searches of the data base (NCBI web page: http://www.ncbi.nlm.nih.gov/sutils/genom_table.cgi) were made using both the left and right flanking sequences as well as the individual stem loop *IS605B* sequences. Identified lipoprotein gene homologs were also scanned for stem loop sequences in their downstream regions.

### Alignment of Nucleotide Sequences

The ClustalW2 program (http://www.ebi.ac.uk/) from the EMBL- European Bioinformatics Institute [23] was used for nucleotide sequence alignments. Parameters were as set on the EMBL-EBI web page. Alignment in figures as well as phylograms are as shown by the ClustalW2 program web pages, with some modifications.

### RNA and DNA Secondary Structure Modeling

The Zuker/Turner mfold program (http://mfold.bioinfo.rpi.edu/cgi-bin/rna-form1.cgi) was used to model RNA sequences and the DNA mfold server (http://mfold.bioinfo.rpi.edu/cgi-bin/dna-form1.cgi) was used for DNA secondary structure modeling [24], [25]. The default parameters shown on the websites were employed.

### Random Mutagenesis

The Stormoff mutagenesis website was used to induce random mutations in stem loop sequences (http://molbiol.ru/eng/scripts/01_16.html). The mutagenesis program was devised by Stothard [26].

## Supporting Information

Figure S1 A comparison of RNA and DNA secondary structure models for the stem loop sequence associated with the lipoprotein gene locus BSV_X04.
(0.04 MB DOC)

## References

[1] Fraser CM, Casjens S, Huang WM, Sutton GG, Clayton R (1997). Genomic sequence of a Lyme disease spirochaete. *Borrelia burgdorferi*.. Nature.

[2] Casjens S, Palmer N, van Vugt R, Huang WM, Stevenson B (2000). A bacterial genome in flux: the twelve linear and nine circular extrachromosomal DNAs in an infectious isolate of the Lyme disease spirochete *Borrelia burgdorferi*.. Mol Microbiol.

[3] Delihas N (2009). Intergenic regions of *Borrelia* plasmids contain phylogenetically conserved RNA secondary structure motifs.. BMC Genomics.

[4] Ronning DR, Guynet C, Ton-Hoang B, Peres ZN, Ghirlando R (2005). Active site sharing and subterminal hairpin recognition in a new class of DNA transposases.. Mol Cell.

[5] Guynet C, Hickman AB, Barabas O, Dyda F, Chandler M (2008). In vitro reconstitution of a single-stranded transposition mechanism of IS608.. Mol Cell.

[6] Barabas O, Ronning DR, Guynet C, Hickman AB, Ton-Hoang B (2008). Mechanism of IS200/IS605 family DNA transposases: activation and transposon-directed target site selection.. Cell.

[7] Mazzone M, De Gregorio E, Lavitola A, Pagliarulo C, Alifano P (2001). Whole-genome organization and functional properties of miniature DNA insertion sequences conserved in pathogenic *Neisseriae*.. Gene.

[8] Delihas N (2007). Enterobacterial small mobile sequences carry open reading frames and are found intragenically –evolutionary implications for formation of new peptides.. Gene Regulation and Systems Biology.

[9] De Gregorio E, Silvestro G, Petrillo M, Carlomagno MS, Di Nocera PP (2005). Enterobacterial repetitive intergenic consensus sequence repeats in yersiniae: genomic organization and functional properties.. J Bacteriol.

[10] De Gregorio E, Silvestro G, Venditti R, Carlomagno MS, Di Nocera PP (2006). Structural organization and functional properties of miniature DNA insertion sequences in *Yersiniae*.. J Bacteriol.

[11] Snyder LA, Cole JA, Pallen MJ (2009). Comparative analysis of two *Neisseria gonorrhoeae* genome sequences reveals evidence of mobilization of Correia Repeat Enclosed Elements and their role in regulation.. BMC Genomics.

[12] Delihas N (2008). Small mobile sequences in bacteria display diverse structure/function motifs.. Mol Microbiol.

[13] De Gregorio E, Abrescia C, Carlomagno MS, Di Nocera PP (2002). The abundant class of nemis repeats provides RNA substrates for ribonuclease III in *Neisseriae*.. Biochim Biophys Acta.

[14] Treangen TJ, Abraham AL, Touchon M, Rocha EP (2009). Genesis, effects and fates of repeats in prokaryotic genomes.. FEMS Microbiol Rev.

[15] Chen Y, Zhou F, Li G, Xu Y (2008). A recently active miniature inverted-repeat transposable element, Chunjie, inserted into an operon without disturbing the operon structure in *Geobacter uraniireducens Rf4*.. Genetics.

[16] Zhou F, Tran T, Xu Y (2008). Nezha, a novel active miniature inverted-repeat transposable element in cyanobacteria.. Biochem Biophys Res Commun.

[17] Ostberg Y, Bunikis I, Bergström S, Johansson J (2004). The etiological agent of Lyme disease, *Borrelia burgdorferi*, appears to contain only a few small RNA molecules.. J Bacteriol.

[18] Lybecker MC, Samuels DS (2007). Temperature-induced regulation of RpoS by a small RNA in *Borrelia burgdorferi*.. Mol Microbiol.

[19] Sittka A, Lucchini S, Papenfort K, Sharma CM, Rolle K (2008). Deep sequencing analysis of small noncoding RNA and mRNA targets of the global post-transcriptional regulator, Hfq.. PLoS Genet.

[20] Schmitz M (2004). Change of RNase P RNA function by single base mutation correlates with perturbation of metal ion binding in P4 as determined by NMR spectroscopy.. Nucleic Acids Res.

[21] McManus CJ, Schwartz ML, Butcher SE, Brow DA (2007). A dynamic bulge in the U6 RNA internal stem-loop functions in spliceosome assembly and activation.. RNA.

[22] Altschul SF, Madden TL, Schäffer AA, Zhang J, Zhang Z (1997). Gapped BLAST and PSI-BLAST: a new generation of protein database search programs.. Nucleic Acids Res.

[23] Labarga A, Valentin F, Andersson M, Lopez R (2007). Web Services at the European Bioinformatics Institute.. Nucleic Acids Res.

[24] Zuker M (2003). Mfold web server for nucleic acid folding and hybridization prediction.. Nucleic Acids Res.

[25] SantaLucia J (1998). A unified view of polymer, dumbbell, and oligonucleotide DNA nearest-neighbor thermodynamics.. Proc Natl Acad Sci U S A.

[26] Stothard P (2000). The Sequence Manipulation Suite: JavaScript programs for analyzing and formatting protein and DNA sequences.. Biotechniques.

