# Supplementary material for: Stem Loop Sequences Specific to Transposable Element IS605 Are Found Linked to Lipoprotein Genes in Borrelia Plasmids
Source: PLoS One. 2009 Nov 20;4(11):e7941. doi: 10.1371/journal.pone.0007941 (PMC2775950; doi:10.1371/journal.pone.0007941)

**Figure S1**

A comparison of RNA and DNA secondary structure models for the stem loop sequence associated with the lipoprotein gene locus BSV_X04. The Zuker/Turner mfold program was used to model RNA sequences and the DNA mfold server was used for DNA secondary structure modeling.

**BSV_X04 RNA Model** **BSV_X04 DNA Model**

delta G= -6.8 kcal/mol delta G= -1.57 kcal/mol


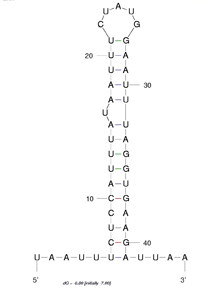

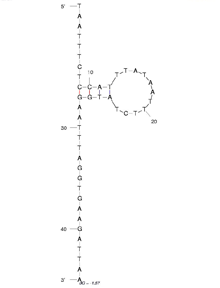

Supplement: Figure S1 — A comparison of RNA and DNA secondary structure models for the stem loop sequence associated with the lipoprotein gene locus BSV_X04. (0.04 MB DOC) [file pone.0007941.s001.doc]
